# Genotyping of *Leptospira* spp. in wild rats leads to first time detection of *L. kirshneri* serovar Mozdok in Serbia

**DOI:** 10.3389/fmicb.2024.1379021

**Published:** 2024-03-28

**Authors:** Vladimir Gajdov, Goran Jokic, Sara Savic, Marina Zekic, Tanja Blazic, Milica Rajkovic, Tamas Petrovic

**Affiliations:** ^1^Scientific Veterinary Institute “Novi Sad”, Novi Sad, Serbia; ^2^Laboratory for Applied Zoology, Institute of Pesticides and Environmental Protection, Belgrade, Serbia; ^3^Laboratory of Experimental Hematology and Stem Cells, Institute for Medical Research, Belgrade, Serbia

**Keywords:** molecular characterization, multilocus sequence typing, sequencing, epidemiology, rat, zoonosis

## Abstract

**Introduction:**

This study aimed to investigate the prevalence and molecular characterization of *Leptospira* species in Belgrade, Serbia, an area where this disease is underexplored. Specifically, the study sought to employ molecular and multilocus sequence typing analyses to fill the gap in understanding the diversity and distribution of *Leptospira* species within the region.

**Methods:**

A comprehensive molecular analysis was conducted on kidney samples obtained from Norway rats (*Rattus norvegicus*) in the urban environment. The study utilized molecular diagnostic techniques including real-time PCR targeting the *lipL32* gene and performing sequence-based typing schemes utilizing *adk, icdA, lipL32, lipL41, rrs2,* and *secY* genes. These methodologies were applied to ascertain the presence and characterize different *Leptospira* species and serovars, respectively.

**Results:**

The findings revealed the presence of two *Leptospira* species and three separate serovars in the Belgrade area. This study identified the presence of *L. kirschneri* serovar Mozdok in Serbia for the first time, a significant discovery previously undocumented in the region. This pioneering investigation sheds light on the molecular diversity and prevalence of *Leptospira* species in Serbia.

**Discussion:**

The study underscores the importance of employing molecular typing methods to gain insights into the epidemiology and characterization of *Leptospira* species. These findings significantly contribute to both local and global perspectives on leptospirosis epidemiology, providing vital insights for the development of effective control strategies and interventions.

**Summary:**

In our recent study, we explored the presence and performed molecular typing of the *Leptospira* species, the bacteria responsible for leptospirosis, in wild rats in Serbia. This was the first time such a study was conducted in the region. Leptospirosis is a serious disease that affects both animals and humans, often transmitted through contact with water contaminated by infected animals. Our focus was on understanding which types of *Leptospira* were present in these animals. Excitingly, we discovered a particular strain of *Leptospira*, known as *L. kirshneri* serovar Mozdok, for the first time in Serbia. This finding is significant because it sheds light on the presence and spread of different *Leptospira* serovars in Serbia. It also raises awareness about the potential health risks associated with this serovar, which was previously unknown in the area. Our work fits into a broader context of disease surveillance and public health. By identifying the types of *Leptospira* present in a specific region, we can better understand the risks to public health and take steps to prevent and control the spread of leptospirosis. This discovery is not just important for scientists studying infectious diseases; it has real implications for public health officials, veterinarians, and anyone concerned with preventing and treating leptospirosis. Our findings highlight the need for ongoing monitoring of *Leptospira* in wildlife and synanthropic fauna, to protect both animal and human health.

## 1 Introduction

Leptospirosis, a zoonotic disease caused by pathogenic spirochaetes of the genus *Leptospira*, is constantly present in some parts of the world and holds significant relevance in both veterinary and public health contexts due to its ability to cross over between humans, domestic animals, wildlife and even environment (water). Reported cases of leptospirosis are global with over one million cases annualy, leading to approximately 60,000 fatalities (Costa et al., 2015). To date, a minimum of 64 distinct *Leptospira* species have been validated worldwide using the average nucleotide identity (ANI) values of their genomes (Vincent et al., 2019). While rats are traditionally known as the primary reservoirs for pathogenic *Leptospira* species, there have been numerous reports on various vertebrate and invertebrate hosts as excreting this pathogen through their urine. Wild and domestic mammals (Arent et al., 2017; Vieira et al., 2017), livestock (Shiokawa et al., 2019; Zhang et al., 2019), amphibians (Dezzutto et al., 2017), reptiles (Rodamilans et al., 2020) and bats (Mateus et al., 2019) also appear to play significant roles in the spread of *Leptospira* sp. Human infections typically result from exposure to soil or water contaminated with *Leptospira*, mostly from the urine of reservoir animals (Adler and de la Peña Moctezuma, 2010). Detecting *Leptospira* through traditional growth on media can be problematic due to their slow growth, making it impractical for timely diagnoses. To address this, molecular diagnostic methods, such as the real-time PCR of the *lipL32* gene, have been developed (Ferreira et al., 2014; Wu et al., 2014). PCR-based amplification of *secY* and *ompL1* genes using species-specific primers and probes has been used to identify *Leptospira* species directly from clinical samples. These assays can identify common pathogenic *Leptospira* species when combined with a *lipL32* assay, including *L. borgpetersenii*, *L. interrogans*, *L. kirschneri*, and *Leptospira noguchii* (Victoria et al., 2008). Furthermore, sequence-based typing schemes utilizing gene targets like 16S rRNA *rrs2*, *secY*, and *lfb1*, or *adk, icdA, lipL32, lipL41, rrs2 and secY* have been developed for *Leptospira* (Ahmed et al., 2006; Morey et al., 2006). For example, a ∼ 435-bp fragment of the *secY* gene shows good phylogenetic discrimination between pathogenic *Leptospira* species. Sequence-based methods can also be applied directly to clinical samples to determine the infecting species and genotype, as well as investigate links between human and animal *Leptospira* infection (Hamond et al., 2015). In Serbia, the presence of pathogenic *Leptospira* sp. has been documented in various animals including small wild mammals (Blagojević et al., 2019), however most of the studies in Serbia have been focused on seroprevalence and seroepidemiological detection of antibodies in samples from cats (Obrenović et al., 2014), dogs (Vojinović et al., 2022) and humans (Svirčev et al., 2009). To the best of our knowledge this is the first study to perform molecular and multilocus sequence typing analysis of *Leptospira* species in Serbia. Moreover, this study revealed the presence of *Leptospira kirshneri* serovar Mozdok in Serbia for the first time.

## 2 Materials and methods

### 2.1 Animal collection

The research was conducted in accordance with ethical principles and was approved by the Ministry of Agriculture, Forestry and Water Management (Republic of Serbia) - Veterinary Directorate (No. 323-07-04943/2020-05/2, 29.05.2020 and 323-07-04155/2023-05/2, 16.05.2023). During 2020, 2021 and 2022, a total of 344 (186 female and 158 male) carcasses of Norway rats (*Rattus norvegicus*) were collected in the broad environs of Belgrade City, predominantly in their urban and suburban 163 habitats. With the aim of collecting material that would be of good quality for further analysis of the presence of bacteria in the tissues, animals were collected by trapping (snap traps with fish mixture with peanut butter and oat flakes as a bait) and carcasses were collected daily and kept at 4°C during transportation to the laboratory where they were measured, followed by necropsy, during which the kidneys were removed and kept at −20°C until further processing and analysis. The average body mass of all used animals (± SD) was 236.97 ± 99.31 g (range 30–498 g). The average body length of the individuals was 201.79 ± 40.09 cm (range 91–374 cm), while the average tail length was 168.14 ± 30.48 cm (range 80–230 cm). Carcasses were collected predominantly in their urban and suburban habitats. The largest number of individuals was collected after the implementation of control measures or the implementation of monitoring measures. The collected carcasses were kept in a freezer at −20°C for a short time, until further processing. During autopsy, the kidneys were separated for further analysis and the morphological data, body weight and sex of the animals were recorded.

### 2.2 DNA extraction, molecular detection, sequencing, and MLST analysis

DNA was extracted from the kidney using the Quick-DNA MiniPrep kit (Zymo Research, USA, Cat. no. D3024) according to manufacturers’ instructions. To validate the extraction processes and all downstream steps, nuclease-free water and DNA extracted from *Leptospira* positive samples were used as negative and positive controls, respectively. DNA extracted from each sample was stored at −20°C until downstream use. To distinguish between pathogenic and non-pathogenic *Leptospira*, we performed qPCR targeting the *lipL32* partial target genes. Specifically, we used primers LipL32F (5′-GGA TCC GTG TAG AAA GAA TGT CGG-3′) and LipL32R (5’-GTC ACC ATC ATC ATC ATC GTC C-3′) to amplify a 101 bp fragment of the *lipL32* gene, which was detected by the probe LipL32P (6-carboxyfluorescein [FAM]-5′-ATG CCT GAC CAA ATC GCC AAA GCT GCG AAA-3’-Black Hole Quencher 1 [BHQ1]) (Wu et al., 2014). An internal control, represented by exogenous DNA added before the extraction phase, representing simultaneously the extraction and PCR amplification control (qPCR Extraction Control RED, Meridian Bioscience, UK) was also included. The qPCR was carried out in a 12 μL reaction mixture containing 3 μL of *Leptospira* spp. genomic DNA, 0.5 μL (concentration of 20 pmol/μL) of forward and reverse primer and probe and 5 μL (concentration of 10 pmol/μL) of FastGene 2x PROBE Universal (Nippon Genetics, Germany) and 2.5 μL of PCR water. All reactions were conducted in duplicates using a 7,500 Fast Real-Time PCR System (Applied Biosystems, ThermoFisher, USA) with the following conditions: initial denaturation at 95°C for 2 min, followed by 45 cycles of denaturation at 95°C for 20 s, and annealing/elongation at 65°C for 50 s. Each PCR test included a negative control (DNA extracted from water) and a positive control (DNA extracted *Leptospira* spp. positive samples). Among the positive samples obtained through qPCR, only those with threshold cycle (Ct) values lower than or equal to 30 underwent further analysis. Specifically, 27 kidney samples and 27 *Leptospira* DNAs were subjected to PCR using a set of primers amplifying *adk*, *icdA*, *lipL32*, *lipL41*, *rrs2* and *secY* partial genes (Table 1) (Ahmed et al., 2006). PCR reagents and their volumes, as well as PCR cycling conditions are shown in Tables 2, 3, respectively. The PCR products were visualized by electrophoresis on a 1.5% agarose gel and examined under UV transillumination.

**Table 1:** Details of gene loci and the corresponding primer sequences used for MLST analysis.

Gene	Locus	Gene size (bp)	Genome position	PCR product (bp)	Size of polymorphic sequence (bp)	Primer sequences 5′-3′
*adk*	LIC12852	564	3,458,298–3,458,861	531	430	F-GGGCTGGAAAAGGTACACAA R-ACGCAAGCTCCTTTTGAATC
*icdA*	LIC13244	1,197	3,979,829–3,981,025	674	557	F-GGGACGAGATGACCAGGAT R-TTTTTTGAGATCCGCAGCTTT
*lipL32*	LIC11352	819	1,666,299–1,667,117	474	474	F-ATCTCCGTTGCACTCTTTGC R-ACCATCATCATCATCGTCCA
*lipL4l*	LIC12966	1,068	3,603,575–3,604,642	520	518	F-TAGGAAATTGCGCAGCTACA R-GCATCGAGAGGAATTAACATCA
*rrs2*	LIC11508	1,512	1,862,433–1,863,944	541	452	F-CATGCAAGTCAAGCGGAGTA R-AGTTGAGCCCGCAGTTTTC
*secY*	LIC12853	1,383	3,458,869–3,460,251	549	549	F-ATGCCGATCATTTTTGCTTC R-CCGTCCCTTAATTTTAGACTTCTTC

**Table 2:** Reagents and volumes.

Reagent	Volume per reaction - μL
DNA template (*Leptospira* DNA)	3
Forward primer	1 (concentration of 20 pmol/μL)
Reverse primer	1 (concentration of 20 pmol/μL)
HotStarTaq master mix	12.5
Sterile water	7.5
Total reaction volume	25

**Table 3:** PCR cycling conditions.

Step	Temperature (°C)	Time	Number of cycles
Initial denaturation	95	15 min	1
Denaturation	95	30 s	35
Annealing	58	30 s	
Extension	72	1 min	
Final extension	72	10	1
Hold	4	∞	1

We purified the amplicons using the GeneJET PCR Purification Kit (ThermoFisher Scientific, USA, cat. no. K0702) and sent them to Macrogen Europe (Amsterdam, Netherlands) for Sanger sequencing. Sequences were analyzed and edited using the Staden package (Staden et al., 2003). Consensus sequence validation was performed against a custom *Leptospira* database using nucleotide blast (BLASTn) (Altschul et al., 1990). Each allele and the allelic profiles (adk-icdA-lipL32-lipL41-*rrs2*-secY) were submitted to the *Leptospira* database (Jolley et al., 2018) for ST assignment.^1^ Sequence similarity of our samples was performed with a custom reference database using Biopython (Cock et al., 2009). All sequences were submitted to NCBI’s GenBank under the following accession numbers: OR920389 - OR920523 for *adk, icdA, lipL32, LipL41* and *secY*, while for *rrs2* OR912477-OR912503.

### 2.3 Statistical analysis

Mean prevalence and confidence intervals (95% CI) for *Leptospira* spp. were determined using the Clopper and Pearson method.

## 3 Results

All 344 samples were analyzed for the presence of pathogenic *Leptospira* species. In kidney tissues, *Leptospira* spp. was detected in a total of 103 out of 344 individuals (29.94, 95% CI: 25.15–35.09) upon amplification by qPCR (Table 4). A total of 27 out of 103 positive samples (with Ct values between 20 and 28) were used in this study. Among all samples, the BLASTn analysis indicated that 26 sequences were affiliated with the *L. interrogans*, and 1 sequence exhibited the closest resemblance to the *L. kirschneri* (with 100% identity). The calculated sequence similarity of our samples with a cutoff value of 95% performed with Biopython was in concordance with the BLASTn results and for some of the samples it was possible to determine the serovar. For the final and definite characterization of our samples we determined the allele profile using the MLST scheme 3 from the PubMLST database.^2^ The MLST analysis yielded the following results: 11 of our samples belong to *L. interrogans* serovar Copenhageni, 12 to *L. interrogans* serovar Icterohaemorrhagiae (all belong to the serogroup Icterohaemorrhagiae) and one to *L. kirschneri* serovar Mozdok (serogroup Pomona). For the rest 3 of our samples, we were only able to determine the taxonomy to the level of species (*L. interrogans*) due to lower sequence quality.

**Table 4:** The average weight (g) ± standard error (MS ± SE) and presence of *Leptospira* ssp. in Norway rat kidney tissues, collected in the period 2020–2021 in the Belgrade, Serbia.

Sex	2020		2021		2022	
	Number of individuals	MS ± SE	Number of individuals	MS ± SE	Number of individuals	MS ± SE
Animals in which the presence of bacteria was not confirmed						
female	23	220.23 ± 13.37	69	243.55 ± 13.76	38	201.89 ± 17.01
male	32	249.37 ± 19.69	49	238.16 ± 12.82	30	233.83 ± 18.29
Animals in which the presence of bacteria was confirmed						
female	16	258.75 ± 22.71	16	250.44 ± 23.08	24	250.42 ± 16.11
male	20	197.25 ± 22.39	15	277.00 ± 22.58	12	253.83 ± 28.58

## 4 Discussion

There is a growing interest in the surveillance of *Leptospira* spp. hosts, and investigations into the prevalence of this pathogen in wildlife and synanthropic faunaacross Europe are on the rise and the significance of rodents as reservoirs for various *Leptospira* serovars has been extensively explored worldwide with various results. It is well-established that wild rats (*Rattus* spp.) are the principal sources of *Leptospira* infection, particularly in urban and peri-domestic environments (Boey et al., 2019). *Rattus norvegicus* is known as the primary host of *L. interrogans* related to the serogroup Icterohaemorrhagiae, which is responsible for the most severe forms of the disease in humans (Haake and Levett, 2014). Our study aimed to examine the circulating *Leptospira* strains in wild rats, utilizing qPCR for initial detection of pathogenic *Leptospira* and MLST analysis for molecular characterization. Our findings confirm that wild rats harbor different serovars of pathogenic *Leptospira* spp. which pose threat to both animal and public health, highlighting the importance of continuous monitoring the presence and diversity of these bacteria in wild animals. The identification of *L. interrogans* serovar Icterohaemorrhagiae and *L. interrogans* serovar Copenhageni aligns with studies from all over Europe: in Sicily the bacteria has been detected in stray dogs and cats (Grippi et al., 2023). In Sardinia authors have reported pathogenic *Leptospira* in hedgehogs, mustelids and wild rodents (Piredda et al., 2021). In Germany, researchers in one study reported that 6% of the tested animals (various small mammals) exhibited positive results for *L. kirschneri* and *L. interrogans* (Obiegala et al., 2016), while *L. interrogans* serovar Icterohaemorrhagiae has been reported in wild rats all over the world (Boey et al., 2019) which is not surprising given that it represents the most common serovar in animals and humans. Additionally, this study relied on the utilization of the *adk, icdA, lipL32, lipL41, rrs2* and *secY* partial genes as a means for molecular typing and differentiating *Leptospira* serovars. The results obtained using these genes align with those obtained from other MLST analyses. Although Leptospirosis has been the subject of numerous studies across various geographical regions, this present investigation in Serbia marks a significant contribution to the field. Prior research in Serbia had mainly focused on seroprevalence and seroepidemiological studies (Svirčev et al., 2009; Obrenović et al., 2014; Vojinović et al., 2022). However, our study distinguishes itself as the first in Serbia to employ molecular and multilocus sequence typing analysis for *Leptospira* species. This unique approach has yielded in discovering the presence of *L. kirshneri* serovar Mozdok in Serbia. This marks the first documented occurrence of this serovar in the country. Similar reports have been documented in Croatia (Majetić et al., 2014). The comprehensive and systematic testing conducted in our study, which included various *Leptospira* genes, facilitated the detailed characterization of positive samples. The sequencing and BLASTn analysis unveiled a predominance of *L. interrogans* in our samples, reinforcing its role as a common pathogenic *Leptospira* species. Further analysis, including the calculation of sequence similarity and allele profiling using the PubMLST database, refined our understanding of the *Leptospira* strains present. Notably, our findings unveiled specific serovars, such as *L. interrogans* serovar Copenhageni and *L. interrogans* serovar Icterohaemorrhagiae, underscoring the diversity of *Leptospira* strains within the Belgrade region. The significance of our discovery of *L. kirshneri* serovar Mozdok in Serbia extends beyond the confines of our study. This novel serovar presence has potential implications for vaccine strategies and epidemiological studies in both human and veterinary epidemiology. Serovars play a crucial role in vaccine formulation, as they determine the specific *Leptospira* strains that the vaccine should target. Consequently, our findings serve as a starting point for a more comprehensive and continuous *Leptospira* sp. surveillance in order to detect new serovars and accordingly adapt management strategies (presently the present vaccine strategies in Serbia include preparations for different animals which contain *L. interrogans* serovar Icterohaemorrhagiae, Canicola, Copenhageni and Bratislava, *L. kirshneri* serovar Grippotyphosa) However, it should be noted that this finding does not imply immediate change in vaccine strategy. To date, in Serbia, this serovar has not been confirmed as the causative agent of leptospirosis, most probably due to the lack of testing in patients infected with this bacterium. On the other hand, multiple research has proved that this serovar is clinically relevant since it has been implicated in human and animal leptospirosis (Cunha et al., 2016; Bertasio et al., 2020a,b). The presence of a novel serovar implies the need further explore and determine the presence of this serovar in other animals and/or humans and perform serological tests to screen for seropositive individuals that may have come into contact with this specific bacterium. Regarding leptospiros epidemiology, the identification of *L. kirshneri* serovar Mozdok opens doors to a more comprehensive understanding of the disease’s distribution and dynamics in the region. The serovar’s presence highlights the complexity of *Leptospira* populations in Serbia and warrants further investigation into its reservoir hosts and transmission dynamics. While the detection of a single serovar in one rat does not definitively establish that species as a reservoir it should be noted that while our findings do not conclusively determine the rat as a carrier, they are supported by other research [that has identified the same strain in additional rats, suggesting a potential role as carriers (Majetić et al., 2014; Obiegala et al., 2016)]. Epidemiological studies must now consider the unique characteristics of this serovar, as it may exhibit distinct patterns of host adaptation and disease transmission. Understanding the prevalence and distribution of this serovar is crucial for developing effective control measures, both in terms of prevention and treatment. Moreover, the discovery emphasizes the importance of continued surveillance and monitoring of *Leptospira* diversity in the region, as new serovars may continue to emerge over time. In conclusion, our study has provided valuable insights into the presence and diversity of *Leptospira* species in Serbia. The discovery of *L. kirshneri* serovar Mozdok serves as a pivotal point for advancing management strategies and epidemiological research in the region. By adapting our approaches to the unique characteristics of this novel serovar, we can better address the challenges of leptospirosis and work toward more effective prevention and control measures for both human and veterinary health. Furthermore, the presence of *L. kirshneri* serovar Mozdok opens new avenues for epidemiological research in Serbia. Further research is essential to unveil the full implications of this discovery and to refine our understanding of the epidemiological landscape in Serbia.

## Data availability statement

The original contributions presented in the study are publicly available. This data can be found here: https://www.ncbi.nlm.nih.gov/nuccore/; OR920389-OR920523, OR912477-OR912503.

## Ethics statement

The animal study was approved by Ministry of Agriculture, Forestry and Water Management (Republic of Serbia) - Veterinary Directorate (No. 323-07-04943/2020-05/2, 29.05.2020 and 323-07-04155/2023-05/2, 16.05.2023). The study was conducted in accordance with the local legislation and institutional requirements.

## Author contributions

VG: Data curation, Investigation, Methodology, Writing – original draft, Writing – review & editing. GJ: Conceptualization, Investigation, Supervision, Writing – original draft, Writing – review & editing. SS: Conceptualization, Formal analysis, Supervision, Writing – review & editing. MZ: Investigation, Methodology, Validation, Writing – original draft. TB: Data curation, Investigation, Resources, Writing – original draft. MR: Data curation, Formal analysis, Writing – original draft. TP: Conceptualization, Investigation, Methodology, Validation, Writing – review & editing.

## Footnotes

^1^http://pubmlst.org/*Leptospira*, accessed in October 2023.

^2^https://pubmlst.org/*Leptospira*

## References

[ref1] Adler B. de la Peña Moctezuma A. (2010). Leptospira and Leptospirosis. Vet. Microbiol. 140, 287–296. doi: 10.1016/j.vetmic.2009.03.012 19345023

[ref2] Ahmed N. Devi S. M. Valverde Mde L. Vijayachari P. Machang'u R. S. Ellis W. A.. (2006). Multilocus sequence typing method for identification and genotypic classification of pathogenic Leptospira species. Ann. Clin. Microbiol. Antimicrob. 5:28. doi: 10.1186/1476-0711-5-28, PMID: 17121682 PMC1664579

[ref3] Altschul S. F. Gish W. Miller W. Myers E. W. Lipman D. J. (1990). Basic local alignment search tool. J. Mol. Biol. 215, 403–410. doi: 10.1016/s0022-2836(05)80360-2 2231712

[ref4] Arent Z. Gilmore C. Ayanz J. M. S.-M. Neyra L. García-Peña F. J. (2017). Molecular epidemiology of Leptospira serogroup Pomona infections among wild and domestic animals in Spain. EcoHealth 14, 48–57. doi: 10.1007/s10393-017-1210-8, PMID: 28213654

[ref5] Bertasio C. Boniotti M. B. Lucchese L. Ceglie L. Bellinati L. Mazzucato M.. (2020a). Detection of new Leptospira genotypes infecting symptomatic dogs: is a new vaccine formulation needed? Pathogens 9:484. doi: 10.3390/pathogens9060484, PMID: 32570803 PMC7350335

[ref6] Bertasio C. Papetti A. Scaltriti E. Tagliabue S. D’Incau M. Boniotti M. B. (2020b). Serological survey and molecular typing reveal new Leptospira serogroup Pomona strains among pigs of northern Italy. Pathogens 9:332. doi: 10.3390/pathogens9050332, PMID: 32365494 PMC7281294

[ref7] Blagojević J. Šekler M. Rajičić M. Pejić B. Budinski İ. Jovanović V.. (2019). The prevalence of pathogenic forms of Leptospira in natural populations of small wild mammals in Serbia. Acta Vet. Hung. 67, 338–346. doi: 10.1556/004.2019.035, PMID: 31549550

[ref8] Boey K. Shiokawa K. Rajeev S. (2019). Leptospira infection in rats: a literature review of global prevalence and distribution. PLoS Negl. Trop. Dis. 13:e0007499. doi: 10.1371/journal.pntd.0007499, PMID: 31398190 PMC6688788

[ref9] Cock P. J. A. Antão T. Chang J. T. Chapman B. Cox C. J. Dalke A.. (2009). Biopython: freely available Python tools for computational molecular biology and bioinformatics. Bioinformatics 25, 1422–1423. doi: 10.1093/bioinformatics/btp163 19304878 PMC2682512

[ref10] Costa F. Hagan J. E. Calcagno J. I. Kane M. J. Torgerson P. R. Martinez-Silveira M. S.. (2015). Global morbidity and mortality of leptospirosis: a systematic review. PLoS Negl. Trop. Dis. 9:e0003898. doi: 10.1371/journal.pntd.0003898, PMID: 26379143 PMC4574773

[ref11] Cunha C. E. Félix S. R. Neto A. C. P. S. Campello-Felix A. Kremer F. S. Monte L. G.. (2016). Infection with *Leptospira Kirschneri* Serovar Mozdok: first report from the southern hemisphere. Am. J. Trop. Med. Hyg. 94, 519–521. doi: 10.4269/ajtmh.15-0505, PMID: 26755566 PMC4775884

[ref12] Dezzutto D. Barbero R. Canale G. Acutis P. L. Biolatti C. Dogliero A.. (2017). Detection of Leptospira Spp. in water turtle (*Trachemys Scripta*) living in ponds of urban parks. Vet. Sci. 4:51. doi: 10.3390/vetsci4040051, PMID: 29056709 PMC5753631

[ref13] Ferreira A. Costa P. Rocha T. Amaro A. Vieira M. L. Ahmed A.. (2014). Direct detection and differentiation of pathogenic Leptospira species using a multi-gene targeted real time PCR approach. PLoS One 9:e112312. doi: 10.1371/journal.pone.0112312, PMID: 25398140 PMC4232388

[ref14] Grippi F. Cannella V. Macaluso G. Blanda V. Emmolo G. Santangelo F.. (2023). Serological and molecular evidence of pathogenic Leptospira Spp. in stray dogs and cats of Sicily (South Italy), 2017–2021. Microorganisms 11:385. doi: 10.3390/microorganisms11020385, PMID: 36838350 PMC9963455

[ref15] Haake D. A. Levett P. N. (2014). Leptospirosis in humans. Curr. Top. Microbiol. Immunol. 387, 65–97. doi: 10.1007/978-3-662-45059-8_5, PMID: 25388133 PMC4442676

[ref16] Hamond C. Pestana C. P. Medeiros M. A. Lilenbaum W. (2015). Genotyping of Leptospira directly in urine samples of cattle demonstrates a diversity of species and strains in Brazil. Epidemiol. Infect. 144, 72–75. doi: 10.1017/s0950268815001363, PMID: 26076668 PMC9507312

[ref17] Jolley K. A. Bray J. E. Maiden M. C. J. (2018). Open-access bacterial population genomics: BIGSdb software, the PubMLST.org website and their applications. Wellcome Open Res. 3:124. doi: 10.12688/wellcomeopenres.14826.1, PMID: 30345391 PMC6192448

[ref18] Majetić Z. Š. Galloway R. L. Sabljić E. R. Milas Z. Perko V. M. Habuš J.. (2014). Epizootiological survey of small mammals as Leptospira Spp. reservoirs in eastern Croatia. Acta Trop. 131, 111–116. doi: 10.1016/j.actatropica.2013.12.009, PMID: 24365042 PMC6374769

[ref19] Mateus J. E. Gómez N. Herrera-Sepúlveda M. T. Hidalgo M. Pérez-Torres J. Cuervo C. (2019). Bats are a potential reservoir of pathogenic Leptospira species in Colombia. J. Infect. Dev. Ctries. 13, 278–283. doi: 10.3855/jidc.10642 32045371

[ref20] Morey R. E. Galloway R. L. Bragg S. L. Steigerwalt A. G. Mayer L. W. Levett P. N. (2006). Species-specific identification of Leptospiraceae by 16S RRNA gene sequencing. J. Clin. Microbiol. 44, 3510–3516. doi: 10.1128/jcm.00670-06, PMID: 17021075 PMC1594759

[ref21] Obiegala A. Woll D. Karnath C. Silaghi C. Schex S. Eßbauer S.. (2016). Prevalence and genotype allocation of pathogenic Leptospira species in small mammals from various habitat types in Germany. PLoS Negl. Trop. Dis. 10:e0004501. doi: 10.1371/journal.pntd.0004501, PMID: 27015596 PMC4807814

[ref22] Obrenović S. Radojičić S. Stević N. Bogunović D. Vakanjac S. Valčić M. (2014). Seroprevalence of cat leptospirosis in Belgrade (Serbia). Acta Vet-Beogr. 64, 510–518. doi: 10.2478/acve-2014-0047

[ref23] Piredda I. Ponti M. N. Palmas B. Noworol M. Pedditzi A. Rebechesu L.. (2021). Molecular typing of pathogenic Leptospira species isolated from wild mammal reservoirs in Sardinia. Animals 11:1109. doi: 10.3390/ani11041109, PMID: 33924303 PMC8069414

[ref25] Rodamilans G. M. Fonseca M. S. Paz L. N. Fernandéz C. C. Biondi I. Lira-Da-Silva R. M.. (2020). *Leptospira Interrogans* in wild *Boa Constrictor* snakes from Northeast Brazil Peri-urban rainforest fragments. Acta Trop. 209:105572. doi: 10.1016/j.actatropica.2020.105572, PMID: 32504590

[ref26] Shiokawa K. Welcome S. Kenig M. Lim B. Rajeev S. (2019). Epidemiology of Leptospira infection in livestock species in saint Kitts. Trop. Anim. Health Prod. 51, 1645–1650. doi: 10.1007/s11250-019-01859-5, PMID: 30877524

[ref27] Staden R. Beal K. F. Bonfield J. K. (2003). The Staden package, 1998. Methods Mol. Biol. 132, 115–130. doi: 10.1385/1-59259-192-2:115, PMID: 10547834

[ref28] Svirčev Z. Marković S. B. Vukadinov J. Stefan-Mikić S. Ružić M. Doder R.. (2009). Leptospirosis distribution related to freshwater habitats in the Vojvodina region (republic of Serbia). Sci. China C Life Sci. 52, 965–971. doi: 10.1007/s11427-009-0124-2, PMID: 19911133

[ref29] Victoria B. Ahmed A. Zuerner R. L. Ahmed N. Bulach D. Quinteiro J.. (2008). Conservation of the S10-spc-α locus within otherwise highly plastic genomes provides phylogenetic insight into the genus Leptospira. PLoS One 3:e2752. doi: 10.1371/journal.pone.0002752, PMID: 18648538 PMC2481283

[ref30] Vieira A. S. Pinto P. S. Lilenbaum W. (2017). A systematic review of leptospirosis on wild animals in Latin America. Trop. Anim. Health Prod. 50, 229–238. doi: 10.1007/s11250-017-1429-y, PMID: 28967042

[ref31] Vincent A. T. Schiettekatte O. Goarant C. Neela V. K. Bernet È. Thibeaux R.. (2019). Revisiting the taxonomy and evolution of pathogenicity of the genus Leptospira through the prism of genomics. PLoS Negl. Trop. Dis. 13:e0007270. doi: 10.1371/journal.pntd.0007270, PMID: 31120895 PMC6532842

[ref32] Vojinović D. Žutić J. Vasić A. Stanojević S. Spalević L. Sapundžić Z. Z. (2022). A serological survey of canine leptospirosis in the City of Belgrade, Serbia. Vet. Glas. 76, 47–55. doi: 10.2298/vetgl210708001v

[ref33] Wu Q. Prager K. C. Goldstein T. Alt D. P. Galloway R. L. Zuerner R. L.. (2014). Development of a real-time PCR for the detection of pathogenic Leptospira Spp. in California Sea lions. Dis. Aquat. Org. 110, 165–172. doi: 10.3354/dao02752, PMID: 25114040 PMC6423441

[ref34] Zhang C. Junjun X. Zhang T. Qiu H. Li Z. Zhang E.. (2019). Genetic characteristics of pathogenic Leptospira in wild small animals and livestock in Jiangxi Province, China, 2002–2015. PLoS Negl. Trop. Dis. 13:e0007513. doi: 10.1371/journal.pntd.0007513, PMID: 31233503 PMC6611636

