# Supplementary material for: Genotyping of Leptospira spp. in wild rats leads to first time detection of L. kirshneri serovar Mozdok in Serbia
Source: Front Microbiol. 2024 Mar 28;15:1379021. doi: 10.3389/fmicb.2024.1379021 (PMC11006980; doi:10.3389/fmicb.2024.1379021)
Supplement: Supplementary file 1 [file Presentation_1.PPTX]

## Slide 1
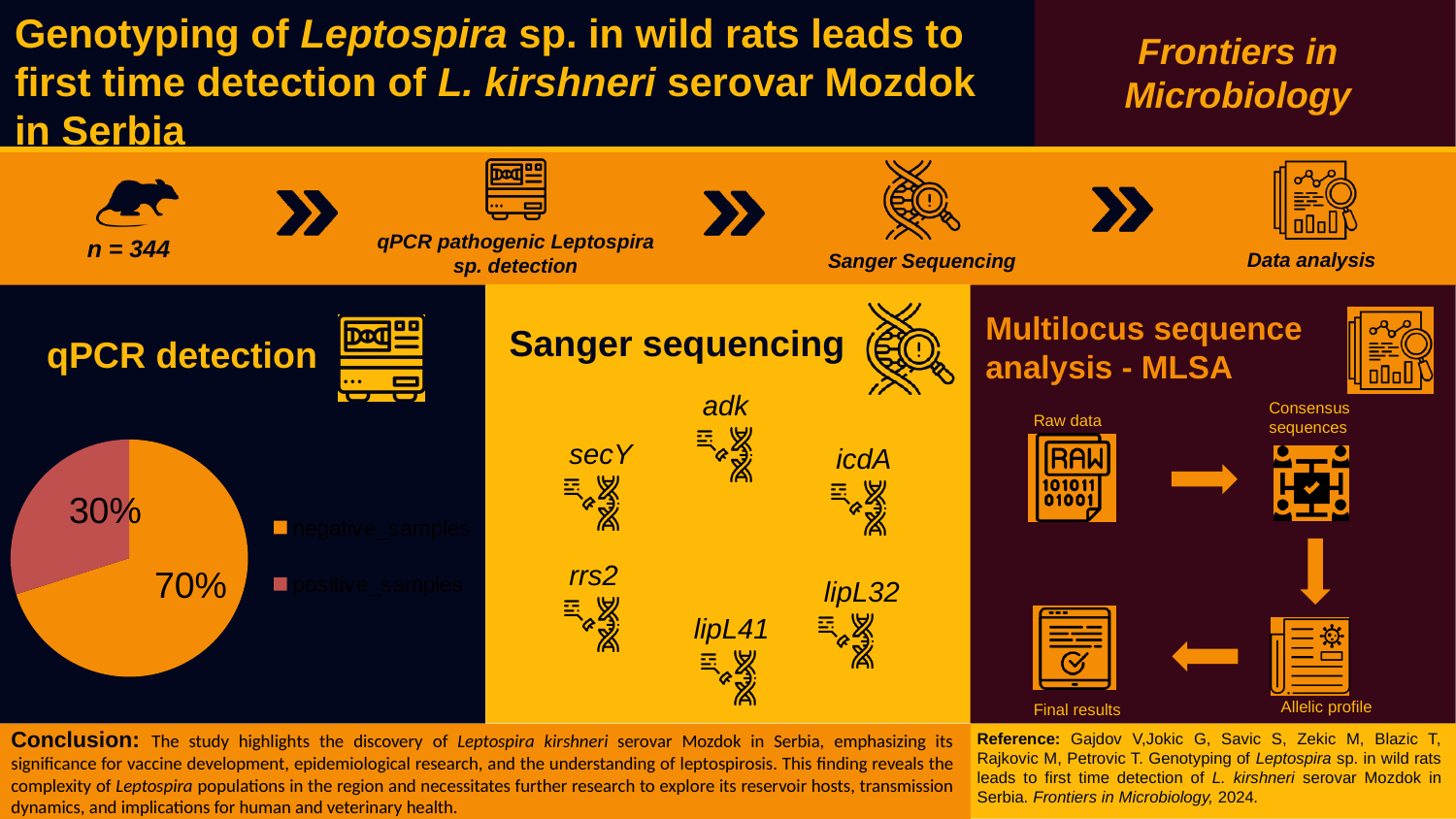

Genotyping of Leptospira sp. in wild rats leads to first time detection of L. kirshneri serovar Mozdok in Serbia
Frontiers in Microbiology
qPCR pathogenic Leptospira sp. detection
n = 344
Data analysis
Sanger Sequencing
Multilocus sequence analysis - MLSA
Sanger sequencing
qPCR detection
### Chart
| Category |
|---|adk
Consensus sequences
Raw data
### Chart
| Category | | |
|---|---|---|
| negative_samples | 241.0 | 241.0 |
| positive_samples | 103.0 | 103.0 |
secY
icdA
30%
rrs2
70%
lipL32
lipL41
Allelic profile
Final results
Conclusion: The study highlights the discovery of Leptospira kirshneri serovar Mozdok in Serbia, emphasizing its significance for vaccine development, epidemiological research, and the understanding of leptospirosis. This finding reveals the complexity of Leptospira populations in the region and necessitates further research to explore its reservoir hosts, transmission dynamics, and implications for human and veterinary health.
Reference: Gajdov V,Jokic G, Savic S, Zekic M, Blazic T, Rajkovic M, Petrovic T. Genotyping of Leptospira sp. in wild rats leads to first time detection of L. kirshneri serovar Mozdok in Serbia. Frontiers in Microbiology, 2024.
